# The Functional Role of the 3′ Untranslated Region and Poly(A) Tail of Duck Hepatitis A Virus Type 1 in Viral Replication and Regulation of IRES-Mediated Translation

**DOI:** 10.3389/fmicb.2018.02250

**Published:** 2018-09-25

**Authors:** Jun-Hao Chen, Rui-Hua Zhang, Shao-Li Lin, Peng-Fei Li, Jing-Jing Lan, Sha-Sha Song, Ji-Ming Gao, Yu Wang, Zhi-Jing Xie, Fu-Chang Li, Shi-Jin Jiang

**Affiliations:** ^1^College of Veterinary Medicine, Shandong Agricultural University, Tai’an, China; ^2^Shandong Provincial Key Laboratory of Animal Biotechnology and Disease Control and Prevention, Tai’an, China; ^3^Department of Basic Medical Sciences, Taishan Medical College, Tai’an, China; ^4^College of Animal Science and Technology, Shandong Agricultural University, Tai’an, China

**Keywords:** DHAV-1, 3′ UTR, poly(A) tail, viral replication, IRES-mediated translation

## Abstract

The duck hepatitis A virus type 1 (DHAV-1) is a member of *Picornaviridae* family, the genome of the virus contains a 5′ untranslated region (5′ UTR), a large open reading frame that encodes a polyprotein precursor and a 3′ UTR followed by a poly(A) tail. The translation initiation of virus proteins depends on the internal ribosome-entry site (IRES) element within the 5′ UTR. So far, little information is known about the role of the 3′ UTR and poly(A) tail during the virus proliferation. In this study, the function of the 3′ UTR and poly(A) tail of DHAV-1 in viral replication and IRES-mediated translation was investigated. The results showed that both 3′ UTR and poly(A) tail are important for maintaining viral genome RNA stability and viral genome replication. During DHAV-1 proliferation, at least 20 adenines were required for the optimal genome replication and the virus replication could be severely impaired when the poly (A) tail was curtailed to 10 adenines. In addition to facilitating viral genome replication, the presence of 3′ UTR and poly(A) tail significantly enhance IRES-mediated translation efficiency. Furthermore, 3′ UTR or poly(A) tail could function as an individual element to enhance the DHAV-1 IRES-mediated translation, during which process, the 3′ UTR exerts a greater initiation efficiency than the poly(A)_25_ tail.

## Introduction

The internal ribosome entry site (IRES) element possesses the function to direct cap-independent internal initiation of protein synthesis; however, the underlying mechanism might differ from that of the canonical cap-dependent translation initiation of the majority of cellular mRNAs (Belsham, 2009). Most eukaryotic mRNAs and many viral RNAs are capped and poly(A) tailed at both termini, thereby regulating translation efficiency individually, or in concert (Hentze, 1997; Sachs et al., 1997). The 5′-cap structure (m7GpppG) can be recognized by translation-initiation factor complex eukaryotic translation initiation factor 4F (eIF4F) for cellular mRNA translation initiation (Gingras et al., 1999). Nevertheless, *Picornavirus* RNA is not 5′ capped and the initiation of viral protein synthesis was termed internal initiation, which relies upon the IRES element within the 5′ UTR (Belsham and Jackson, 2000). As the only member of the novel genus *Avihepatovirus*, family *Picornaviridae*, DHAV is a positive, single-stranded, non-enveloped RNA virus with a ∼7700-nucleotide genome that contains one open reading frame (ORF) encoding three structural proteins and nine non-structural proteins and flanked by a 5′ UTR and a 3′ UTR with a poly(A) tail (Kim et al., 2006). Excluding the poly(A) tail, the 3′ UTR of DHAV type 1 (DHAV-1) is 314 nucleotides in length, the longest among all picornaviruses, and comprises three stem-loops (Ding and Zhang, 2007). Based on the secondary structure characteristics and biological properties, *picornaviral* IRES elements have been classified into five groups (Borman et al., 1995; Hellen and Wimmer, 1995; Borman and Kean, 1997; Pisarev et al., 2004; Yu et al., 2011; Sweeney et al., 2012; Asnani et al., 2015). The DHAV-1 IRES element is categorized as a type IV IRES, which is found essential for internal translation initiation (Pan et al., 2012).

The untranslated region of *Picornaviruses* played important roles in viral genome replication, translation and infectivity. For example, deletion, or substitution of the *aphthovirus* 3′ UTR abrogates the infectivity and virus replication (Saiz et al., 2001); By interfering with the viral polymerase and 5′ UTR, silent mating type information regulation 2 homolog 1 (SIRT1) significantly inhibited viral genome replication and RNA translation of Enterovirus 71 (EV71) (Han et al., 2016); The 3′ UTR determines the virulence of FMDV through regulation of IRES activity (Garcia-Nunez et al., 2014); Besides, it has been demonstrated that RNA structural domains in non-coding regions of the FMDV genome trigger innate immunity in porcine cells and mice (Rodriguez-Pulido et al., 2011). Because viral negative-strand RNA synthesis requires both 3′ and 5′ UTRs, the viral genome template is supposed to form a transient circular conformation during the negative-strand RNA synthesis (Kaku et al., 2002; Svitkin et al., 2007). In *picornaviruses*, the poly(A) tail stimulates IRES-mediated translation of reporter mRNAs, such as poliovirus (PV), human rhinovirus (HRV), and encephalomyocarditis virus (EMCV) (Bergamini et al., 2000; Michel et al., 2001; Svitkin et al., 2001; Chard et al., 2006; Bakhshesh et al., 2008; de Breyne et al., 2008). Poly(A) tail also plays an important role in viral replication by serving as the template for 3D^pol^-catalyzed uridylylation, which initiates viral RNA replication (Paul et al., 1998; Willcocks et al., 2011). Curtailing the poly(A) tail can destabilize cellular mRNAs and result in their degradation through 5′ and 3′ exonucleases (Bernstein et al., 1989; Ford et al., 1997; Svitkin et al., 2007). Moreover, negative-strand RNA synthesis is significantly inhibited if the poly(A) tail contains less than eight adenosine nucleotides (Herold and Andino, 2000). Among PVs, the length of the 3′ poly(A) tail is closely associated with replication and infectivity of PV RNA (Sarnow, 1989; Herold and Andino, 2001. PV RNA with shortened or obliterated poly(A) tails has weak infectivity or retarded replication (Khaleghpour et al., 2001; Karim et al., 2006). Shortening or removal of the 3′ poly(A) tail is expected to inhibit the viral-replication process and result in a decline in negative-strand RNA synthesis by inhibiting 3D^pol^-catalyzed uridylylation, due to the insufficient length of the poly(A) tail for binding to 3D^pol^ (Svitkin and Sonenberg, 2007). The current study shows that 3′UTR of DHAV-1 is responsible for the binding of RNA-dependent RNA polymerase, 3D^pol^ (Zhang et al., 2017), while little is known about the functional role of the DHAV-1 3′ UTR and poly(A) tail in both viral genome replication and IRES-mediated translation efficiency. In this study, we investigated the functional role of the 3′ UTR or poly(A) tail in viral replication and IRES-mediated translation efficiency through RNA-launched infectious clone and monocistronic reporter system.

## Materials and Methods

### Virus, Cell and Antibody

DHAV-1 virulent strain LY0801 (GenBank no. FJ436047) was isolated in 2008 from an outbreak of severe DVH in Shandong province, China (Gao et al., 2012). BHK-21 cells, HEK 293T cells, and duck embryo fibroblast (DEF) cells were cultured at 37°C in 5% CO_2_ in Dulbecco’s modified Eagle medium (DMEM) supplemented with 10% fetal bovine serum (FBS), 100 U/mL penicillin, and 100 μg/mL of streptomycin sulfate. For liposome-mediated transfection, cells were seeded into 24-well plates and grown in DMEM with 10% FBS and antibiotics to 90% confluence. The anti-DHAV-1 monoclonal antibody (mAb) 4F8 which could recognize the epitope “_75_GEIILT_80_” lying in VP1 of DHAV-1 was stored in our lab (Zhang et al., 2015). Horseradish peroxidase (HRP)- conjugated goat anti-mouse antibody was obtained from Abcam (Cambridge, MA, United States).

### Plasmids Construction

**FIGURE 1:**
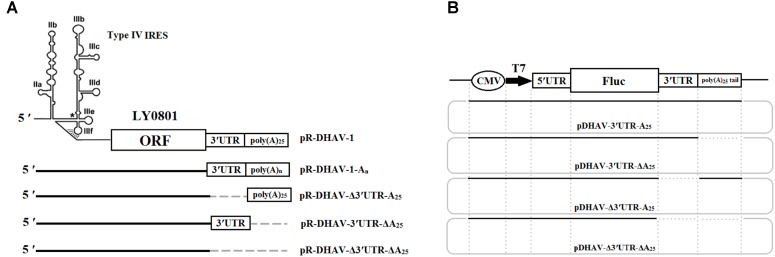
Plasmids construction. **(A)** RNA-launched infectious clones absent/present poly(A) tail or 3′ UTR were established. Besides, the mutated RNA-launched infectious clones possessed various length of poly(A) tail were also constructed and named pR-DHAV-A_n_ (*n* = 0, 5, 10, 15, 20, 25, 30, and 40). **(B)** Construction of the DHAV-1 monocistronic reporter system. The plasmid pDHAV-3′UTR-A_25_ contains the following elements 5′ to 3′ in a pcDNA3.1/V5-His A vector: the CMV immediate early promoter, a T7 promoter, the entire DHAV-1 5′ UTR (nucleotides 1–626, strain LY0801), and the *FLuc* gene and the entire DHAV-1 3′ UTR followed by a poly(A) tail containing 25 adenines. pDHAV-Δ3′UTR-ΔA_25_, or pDHAV-3′UTR-ΔA_25_, or pDHAV-Δ3′UTR-A_25_ were obtained from pDHAV-3′UTR-A_25_ to remove 3′ UTR plus poly(A) tail, or poly(A) tail, or 3′ UTR, respectively. The mutated monocistronic reporter plasmids possessed various length of poly(A) tail was constructed and named as pDHAV-3′UTR-A_n_ (*n* = 0, 5, 10, 15, 20, 25, 30, and 40).

The DHAV-1 RNA-launched infectious clone pR-DHAV-1 was previously constructed based on LY0801 strain genome and stored in our lab (Chen et al., 2017). A series of recombinant plasmids possessed various length of poly(A) tail were established based on the plasmid pR-DHAV-1 and were named as pR-DHAV-1-A_n_ (*n* = 0, 5, 10, 15, 20, 25, 30, and 40) (**Figure 1A**). Three mutated recombinant plasmids absent of 3′ UTR and/or poly(A) tail were established on the base of pR-DHAV-1 and were named pR-DHAV-Δ3′UTR-A_25_, or pR-DHAV-3′UTR-ΔA_25_, or pR-DHAV-Δ3′UTR-ΔA_25_, respectively (**Figure 1A**). The mutated RNA-launched infectious clone pR-DHAV-R3′UTR-A_25_ was established by replacing the 3′ UTR with its reverse complementary sequence (**Figure 1A**). The monocistronic reporter plasmid pDHAV-3′UTR-A_25_ contained the following elements from 5′ to 3′ in a pcDNA^TM^3.1/V5-His A vector: the cytomegalovirus (CMV) immediate early promoter, a T7 promoter, the entire 5′ UTR (nucleotides 1–626), the *Firefly luciferase* (*Fluc*) gene, and the entire DHAV-1 3′ UTR, followed by a poly(A) tail containing 25 adenosine nucleotides (**Figure 1B**). The mutated reporter plasmids absent of poly(A) tail, or 3′ UTR, or the both, were established and named as pDHAV-3′UTR-ΔA_25_, or pDHAV-Δ3′UTR-A_25_, or pDHAV-Δ3′UTR-ΔA_25_, respectively (**Figure 1B**). The recombinant monocistronic reporter plasmids possess various lengths of poly(A) tails (0, 5, 10, 15, 20, 25, 30, and 40 adenosine nucleotides) were constructed based on pDHAV-3′UTR-A_25_ and were named pDHAV-1-A_n_ (**Figure 1B**).

### *In vitro* Transcription

The recombinant plasmids were linearized by digestion with restriction endonuclease HindIII and XhoI and then gel purified according to manufacturer instructions (Omega Bio-Tek, Norcross, GA, United States). The gel-extracted products were quantified using a spectrophotometer (Eppendorf, Cambridge, United Kingdom) and then used for *in vitro* transcription using the T7 RiboMAX Express large-scale RNA production system (Promega, Madison, WI, United States). RNase-free DNase I (TaKaRa, Dalian, China) was added to the *in vitro* transcription products and incubated at 37°C for 15 min to digest the residual DNA template. RNA was purified using RNeasy kits (QIAGEN, Hilden, Germany). The integrity of the transcribed products was determined through PCR amplification with Forward primer: 5′-TGT AAT GGT TCC ATG TGT TCA TCT GGC TAA-3′ and Reverse primer: 5′-TGT GTG GGA CTC GAC CAG CCG CGA CC-3′, and the negative PCR amplification results showed that the template DNA was completely digested. The PCR was done as the following cycling conditions: 7 min at 95°C; 32 cycles of denaturation at 95°C for 50 s, annealing at 55°C for 50 s, and elongation at 72°C for 1 min; 10 min at 72°C.

### Transfection

For liposome-mediated transfection, cells (∼2 × 10^5^) were seeded into 24-well plates and grown in DMEM with 10% FBS and antibiotics to 90% confluence. For RNA transfection, 0.8 μg of the *in vitro* transcribed RNA was diluted in 50 μL opti-MEM and mixed with 2 μL of RNAfectin TRANSfection Reagent (TIANGEN, Beijing, China) in 50 μL opti-MEM after a 5-min of incubation at 25°C. The mixture was added to the 24-well plate after replacing the medium with 100 μL opti-MEM. After the indicated times, the medium was aspirated, and the cells were washed three times with phosphate-buffered saline (PBS containing 8.1 mM Na_2_HPO_4_, 1.5 mM KH_2_PO_4_, 140 mM NaC1, and 3.0 mM KC1). The luciferase activity was measured through Dual-Luciferase^®^ Reporter Assay System (Promega). After aspirating the PBS, 100 μL of passive lysis buffer was added to each well, and the plate was placed on a horizontal table for 5 min at 25°C. Cell lysates were collected and centrifuged at 13,000*g* for 5 min at 4°C, and the pellet was discarded. Luciferase activity was measured in 50-μL aliquots of lysed cells using a Berthold luminometer (GloMax 20/20; Promega).

### Northern Blot Measurement

The DEFs (∼2 × 10^5^) that transfected with monocistronic reporter RNAs or *in vitro* transcribed RNA-launched infectious clones were harvested at 4 hpt. The viral genome RNA of strain LY0801 was transfected into DEFs under same process as positive control. The resulting lysates were then centrifuged at 12,000 Revolutions per Minute (r.p.m.) for 5 min and total RNA was extracted with Total RNA Kit (Omega Bio-Tek) and resuspended in 20 ml of TE. All the total RNA samples were electrophoresed on a 1.5% formaldehyde-agarose gel, transferred to Immobilon-Ny + Membrane (merck Millipore, Darmstadt, German) at 10–15 V (90 min) using *Trans-*Blot SD Semi-Dry Transfer Cell (Bio-Rad), and then cross-linked on the blots by UV illumination with Funa-UV-Crosslinker (Funakoshi, Tokyo, Japan). RNA probes for strand-specific detection of DHAV-5′UTR were prepared by cloning the *Xho* I–*Sma* I fragment (nt 81–351) of LY0801 strain into pBluescript II KS ( + ) (Stratagene, La Jolla, CA, United States) behind the T7 promoter. After linearization of the recombinant plasmids with *Sma* I, the inserts were transcribed *in vitro* using using DIG Northern Starter Kit (Roche, Indianapolis, IN) according to the manufacturer’s instructions. Probe detection was performed using DIG Northern Starter Kit (Roche) according to manufacturer instructions. Briefly, membranes were prehybridized for at least 1 h in UltraHyb (Ambion, Austin, TX, United States) at 68°C, and then were hybridized at 68°C for 16 h with digoxigenin (DIG)-labeled RNA probes. After washing with washing buffer (0.1 M maleic acid, 0.15 M NaCl at pH 7.5, 0.3% Tween 20 [v/v]), membranes were immersed in a blocking solution for 60 min and incubated with anti-digoxigenin-AP Fab fragment (1:10,000 dilution). DIG-bound RNA was visualized with CDP-Star according to the manufacturer’s protocol (Roche). Chemifluorescence was detected by exposing the membrane to X-ray film for 15–25 min at 15–25° C.

### Western Blot Assay

Following transfection, cell lysates were subjected to SDS-PAGE on a 12% polyacrylamide gel, and the separated proteins were electroblotted onto a polyvinylidene fluoride (PVDF) membrane (Thermo Fisher Scientific). Thereafter, the PVDF membrane was blocked with 5% non-fat milk in Tris-buffered saline with Tween 20 (TBST, 500 ml NaCl, 0.05% Tween 20, 10 mM TRIS-HCl, pH 7.5) for 1 h. Thereafter, the membrane was incubated with anti-DHAV-1 mAb 4F8 (1:500) at 4°C for 8 h. The membrane was washed four times with TBST and incubated with a HRP-conjugated goat anti-mouse antibody (1:3000) at 4°C for 4 h. The PVDF membrane was then visualized with hydrogen peroxide and 3,3′-diaminobenzidine tetrahydrochloride (Sigma-Aldrich, St. Louis, MO, United States).

### RT-qPCR

Total RNA was extracted according to manufacturer instructions of Total RNA Kit (Omega Bio-Tek). The extracted RNA was immediately quantified by RT-qPCR, as described previously (Lin et al., 2016). Using the primers RT-qPCR-F/R and the DHAV-1-Probe Cy5-5′-ATG CCA TGA CAC TAT CTC ATA TGA GTC AGC-3′-BHQ-2, a TaqMan real-time RT-PCR assay for quantitative detection of DHAV-1 was conducted in a total volume of 25 μL and containing 12.5 μL 2 × One-step RT-qPCR buffer (with ROX), 0.4 μM of forward and reverse primers, 0.2 μM of the probe, 0.9 μL EnzyMix, and 2 μL of template RNA. The reaction cycles were as follows: 95°C denaturation for 5 min, followed by 40 cycles at 95°C for 15 s, annealing at 60°C for 45 s, with fluorescence measured at every annealing step. Non-template control samples were included in each reaction. Viral RNA copies were calculated using the formula X = 6.7 × 10^(40.812-y)/3.285^, where X represents a standard of viral copies, and y represents a standard of values derived from one-step real-time PCR.

### Statistical Analyses

All experiments were performed at least three times with at least three biological replicates. Statistical significance was evaluated by SAS software (v.8.2; SAS Institute, Inc., Cary, NC, United States) to mean ± SD using the Student *t*-test (^∗^*P <* 0.05, ^∗∗^*P <* 0.01, ^∗∗∗^*P <* 0.001).

## Results

### Effect of Poly(A) Tail and 3′ UTR on RNA Stability

**FIGURE 2:**
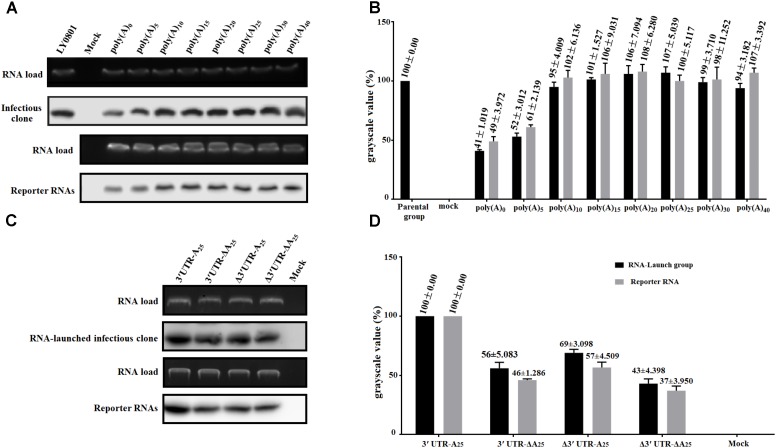
Effects of poly(A) tail length and 3′ UTR on RNA stability. DEFs transfected with 0.8 μg of the *in vitro* transcribed RNA were collected at 4 hpt and were immediately used for total RNA extraction. The samples were analyzed by 1.5% formaldehyde-agarose gel electrophoresis and autoradiography. Digoxigenin (DIG)-labeled RNA probes (based on 81–351 of LY0801) was used to in this assay. The gels show the amount of input RNA remaining at 4 hpt. **(A)** The remaining amount of pR-DHAV-1-A_n_ or pDHAV-1-A_n_ was measured at 4 hpt. **(B)** The grayscale value of represent the mean ± standard deviation of three replicate experiments for poly(A) n group. The grayscale value of parental group was set as 100%. **(C)** The remaining amount of complete/mutated RNA-launched infectious clone or reporter RNA was measured at 4 hpt. The upper panel shows the ethidium bromide staining of the agarose gel loaded with aliquots of 800 ng RNAs. **(D)** The average grayscale values of three replicate experiments for each group. The grayscale value of pR-DHAV-1 or pDHAV-3′UTR-A_25_ was set as 100%.

With the aim to measure the functional role of poly(A) tail and 3′ UTR in viral translation, RNA-launched infectious clone with/without poly(A) tail or 3′ UTR (**Figure 1A**) and monocistronic reporter plasmids with/without poly(A) tail or 3′ UTR (**Figure 1B**) were constructed. Besides, to measure the length of poly(A) tail on viral translation, a series of RNA-launched infectious clone or recombinant monocistronic reporter plasmids with various length of poly(A) tail (A = 0, 5, 10, 15, 20, 25, 30, and 40) (**Figures 1A,B**) were established. To measure the mRNA stability, DEFs transfected with *in vitro* transcribed RNA of complete/mutated RNA-launched infectious clone, or complete/mutated monocistronic reporter RNA were harvested at 4 hpt and were immediately used for total RNA extraction. The extracted RNA was then used for northern blot analysis as previously described. The result of the assay showed that there was no significant difference in relative stability of the viral RNA with 10–40 adenines and the full-length LY0801 RNA (**Figure 2A**), while the viral RNA possessing only 0–5 adenines showed 47–59% decrement on relative stability. Similarly, the RNA transcripts from the reporter plasmids possessing poly(A)_10-40_ tail showed equally higher stability compared to poly(A)_0-5_ groups (**Figure 2A**). Based on the conclusion that the viral RNA of LY0801 strain possess equal relative stability compared to poly(A)_10-40_ RNAs, relative stability of the mutated viral RNA or reporter RNA absent 3′ UTR, or poly(A) tail, or both 3′ UTR and poly(A) tail were measured. The result showed that absence of 3′ UTR or poly(A) tail led to significant decrement on relative stability in both viral RNA and reporter RNA groups, and the decrement was magnified when both 3′ UTR and poly(A) tail were removed (**Figure 2B**).

### The Poly(A) Tails Varying From 10 to 40 Are Indispensible for DHAV-1 Genome Replication

**FIGURE 3:**
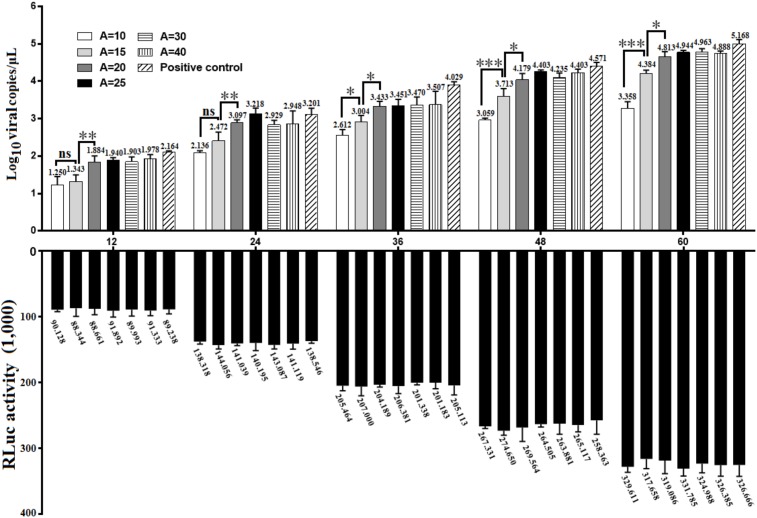
Growth characteristics of DHAV-1 with various length of poly(A) tail. 0.8 μg of *in vitro* transcribed product of pR-DHAV-3′UTR-An were transfected into DEFs, cell lysates were collected from 12 to 60 hpt and were immediately used for total RNA extraction. Viral growth characteristics were measured by RT-qPCR as previously described. Bars represent the mean ± S.D of three replicate experiments for each group. 0.1 μg of pGMLR-TK plasmid was co-transfected with RNA samples, and the RLuc activity was measured at each detection point. ^∗^*P <* 0.05, ^∗∗^*P <* 0.01, ^∗∗∗^*P <* 0.001, ns represent *P >* 0.05.

To investigate whether there was a gradual linear increase in viral replication correlated to the length of poly(A) tail, the *in vitro* transcribed products of pR-DHAV-1-A(n) were transfected into DEFs, cell lysates were collected from 12 to 60 hpt and the viral copy numbers were measured through RT-qPCR. The results showed that DHAV-1 replication was strongly influenced by the number of adenine bases (**Figure 3**). Little or no replication was observed in the poly(A)_0_ and poly(A)_5_ groups (data not shown). When poly(A) tail length varied from 20 to 40 nucleotides, the virus could replicate in a time-dependent manner and no significant difference (*P >* 0.05) was observed at each time point. The highest viral copy number was observed in the poly(A)_25_ group among all time points, with one exception at 36 hpt. No significant difference (*P >* 0.05) on viral copy number was observed at 12 and 24 hpt when the poly(A) tail length increased from 10 to 15 nucleotides, however, the viral copy number increased ≥ 14-fold (*P <* 0.01) at 36 hpt, ≥ 19-fold (*P <* 0.001) at 48 hpt and ≥ 20-fold (*P <* 0.001) at 60 hpt. Besides, the viral copy number exhibited significant increment (*P <* 0.001 at 12 and 24 hpt, and *P <* 0.01 at 36 to 60 hpt) when poly(A) tail length increased from 15 to 20 (**Figure 3**). Based on the RNA stability results that the RNA transcripts of pR-DHAV-1-A_10_, A_15_ and A_20_ shared similar stability (**Figure 2A**), the observed increase in viral replication was the result of a direct effect of poly(A) tail length but not an indirect consequence of a change in RNA stability. The viral copy number in poly(A)_0_ and poly(A)_5_ group showed extremely low levels at each detection point, indicating that the degradation on RNA stability in poly(A)_0_ and poly(A)_5_ group could be one of the reasons for its inability to proliferate. In order to measure the RNA transfection efficiency among each group, 0.1 μg of pGMLR-TK plasmid was co-transfected with RNA samples, and the results showed that RLuc expression level exhibited no significant difference at each detection point.

### DHAV-1 3′ UTR Facilitates the Viral RNA Replication

**FIGURE 4:**
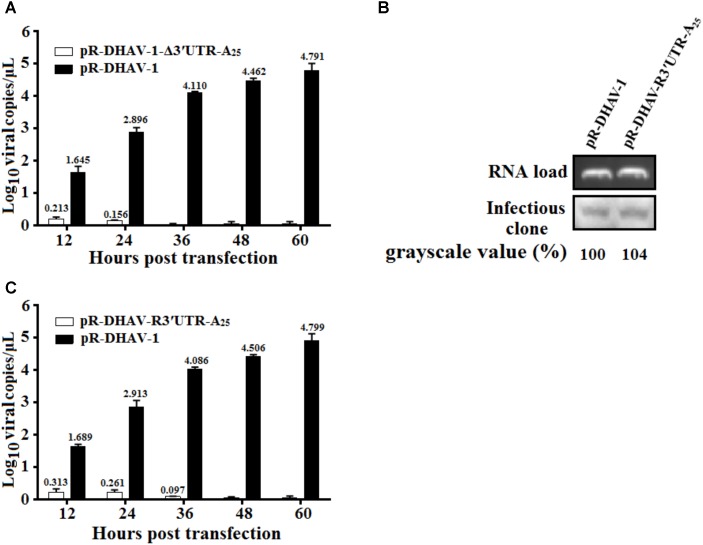
The 3′ UTR is essential for viral genome replication. **(A)** 0.8 μg of the *in vitro* transcribed products of pR-DHAV-1 or pR-DHAV-Δ3′UTR-A_25_ were transfected into DEFs, cell lysates were collected from 12 to 60 hpt and viral copy number was measured by RT-qPCR. **(B)** Equal amount of *in vitro* transcribed product of pR-DHAV-1 or pR-DHAV-R3′UTR-A_25_ were transfected into DEFs, cell lysates were collected at 4 hpt to measure the remaining amount of viral RNAs. **(C)** Equal amount of 0.8 μg of the *in vitro* transcribed products of pR-DHAV-1 or pR-DHAV-R3′UTR-A_25_ were transfected into DEFs to measure viral growth characteristics as previously described. Bars represent the means ± standard deviations of three replicate experiments.

To gain insight into the function of the 3′ UTR during viral replication of DHAV-1, same amount of the *in vitro* transcribed RNAs of pR-DHAV-1 or pR-DHAV-Δ3′UTR-A_25_ were transfected into DEFs. The DHAV-1 viral copy number in cell lysates were determined from 12 to 60 hpt using RT-qPCR. The results showed that the viral copy number increased consistently and significantly (**Figure 4A**) in a time-dependent manner in the pR-DHAV-1 group, whereas no replication increment was observed in the 3′UTR-deletion group (**Figure 4A**). Deletion of 3′ UTR resulted in a 31% decrease in RNA stability at 4 hpt, indicating that the replication deficiency could be caused by the instability of viral RNA. To exclude the effect of the RNA degradation, the *in vitro* transcribed product of mutated infectious clone pR-DHAV-R3′UTR-A_25_ was transfected into DEFs to conduct RNA stability measurement, and the result showed that pR-DHAV-1 shared similar RNA stability with pR-DHAV-R3′UTR-A_25_ (**Figure 4B**). Based on that, viral growth characteristics were measured through RT-qPCR. The results showed that exchange of 3′ UTR into the reverse complementary sequence still led to significant decrement on viral genome numbers (**Figure 4C**). These data indicated that 3′ UTR of DHAV-1 was indispensable for viral RNA replication.

### The 3′ UTR and Poly(A) Tail Potently Stimulate IRES-Mediated Translation

**FIGURE 5:**
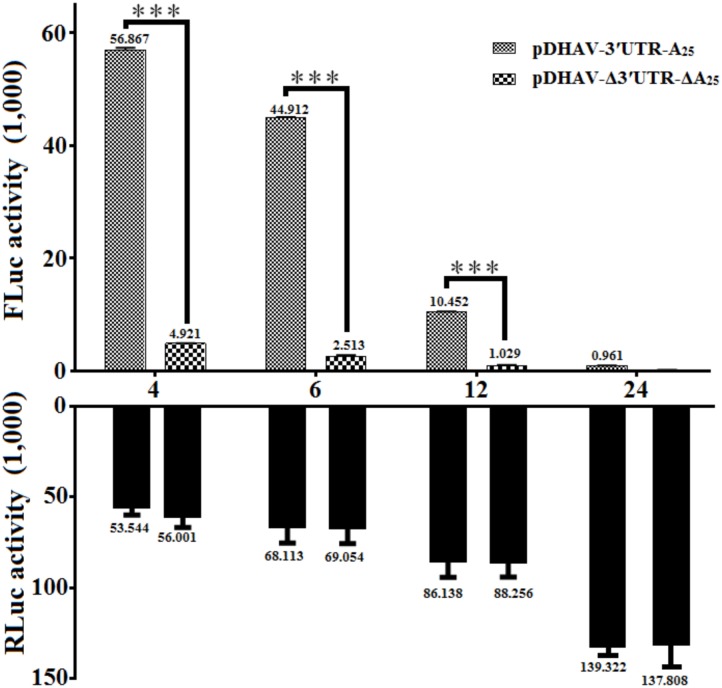
The effect of the DHAV-1 3′ UTR plus the poly(A)_25_ tail on IRES-mediated translation efficiency. 0.8 μg of of the *in vitro* transcribed products of the linearized plasmids pDHAV-3′UTR-A_25_ and pDHAV-Δ3′UTR-ΔA_25_ were co-transfected into DEFs with 0.1 μg of pGMLR-TK plasmid, and the FLuc/RLuc activities were measured from 4 to 12 hpt. Columns and bars represent the means and standard deviations, respectively, of three independent transfections. ^∗^*P <* 0.05; ^∗∗∗^*P <* 0.001.

To measure the possible role of 3′ UTR and poly(A) tail during viral translation, equal quantities of 0.8 μg of the *in vitro* transcribed products of the linearized plasmids pDHAV-3′UTR-A_25_ or pDHAV-Δ3′UTR-ΔA_25_ (**Figure 1B**) were transfected into DEFs, and the FLuc activities were measured from 4 to 24 hpt. To measure the RNA transfection efficiency among all groups, 0.1 μg of pGMLR-TK plasmid was co-transfected with the reporter RNAs, and the RLuc activity was also detected. The results showed that the RLuc activity among all groups exhibited no significant difference. Although the deletion of 3′ UTR and poly(A) tail could result in a 63% decrease in RNA stability (**Figure 2B**), FLuc activity of the 3′ UTR and poly(A)_25_ tail-containing group was much higher (10–38-fold, *P <* 0.001) than that in the group without the 3′ UTR and poly(A) tail (**Figure 5**), indicated 3′ UTR and poly(A) tail potently stimulate IRES-mediated translation.

### The 3′ UTR and Poly(A) Tail Individually Affect DHAV-1 IRES-Mediated Translation Efficiency

**FIGURE 6:**
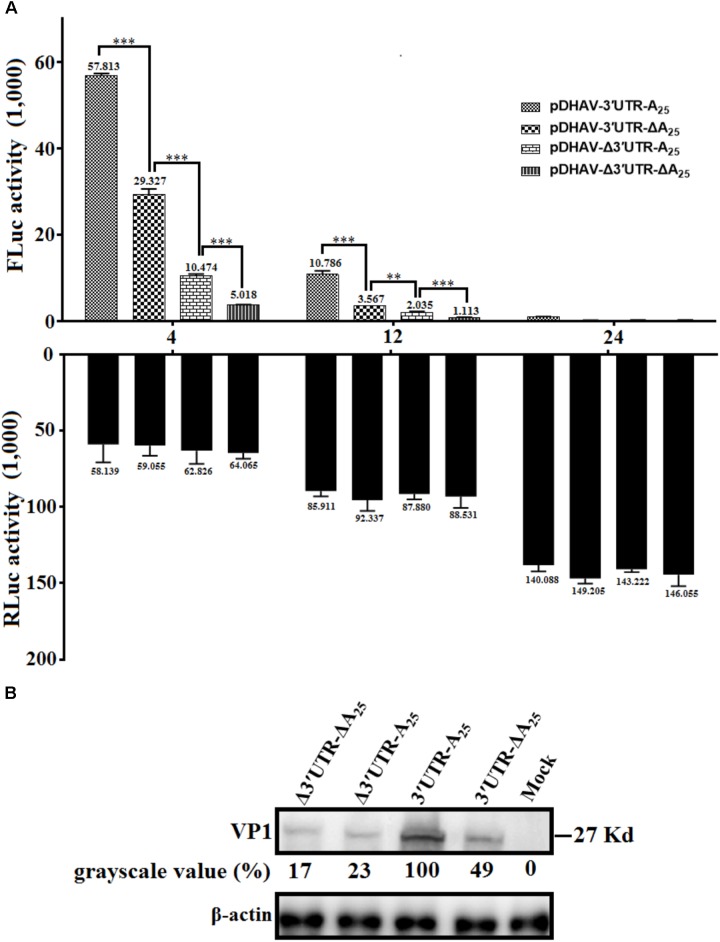
The 3′ UTR and poly(A) tail function as individual elements to stimulate IRES-mediated translation. **(A)** The *in vitro* transcribed products of plasmid pDHAV-3′UTR-ΔA_25_ or pDHAV-Δ3′UTR-A_25_ were transfected into DEFs, and reporter RNAs of pDHAV-3′UTR-A_25_ or pDHAV-Δ3′UTR-ΔA_25_ were used as positive or negative controls, respectively. The stimulation values between 3′ UTR-containing RNAs and poly(A)-containing RNAs were compared. 0.1 μg of pGMLR-TK plasmid was co-transfected with reporter RNAs, and the RLuc activity was measured at each detection point. Bars represent the means ± standard deviations of three replicate experiments. ^∗∗∗^*P <* 0.001, ^∗∗^*P <* 0.01. **(B)** After transfection of pR-DHAV-1-3′UTR-ΔA_25_ or pR-DHAV-1-Δ3′UTR-A_25_ into DEFs, cell lysates were collected at 4 hpt and used for western blot analysis, with pR-DHAV-1 and pR-DHAV-1-Δ3′UTR-ΔA_25_ used as controls. The anti-DHAV-1 monoclonal antibody 4F8 (dilution: 1:500) and HRP-labeled goat anti-mouse antibody (dilution: 1:3000) were used for western blot analysis.

To determine whether the 3′ UTR or poly(A) tail alone could affect IRES-mediated translation efficiency of DHAV-1, equal quantities of 0.8 μg of the *in vitro* transcribed products of the linearized plasmids pDHAV-3′UTR-A_25_, or pDHAV-Δ3′UTR-A_25_, or pDHAV-3′UTR-ΔA_25_, or pDHAV-Δ3′UTR-ΔA_25_ (**Figure 1B**) were co-transfected into DEFs with 0.1 μg of pGMLR-TK plasmid, and FLuc/RLuc activities were measured at different times post transfection. With similar transfection efficiency (measure by RLuc activity), considerably lower luciferase activity in the pDHAV-3′UTR-ΔA_25_ and pDHAV-Δ3′UTR-A_25_ groups was observed compared with that in the pDHAV-3′UTR-A_25_ group at 4 and 12 hpt. The FLuc activities in pDHAV-Δ3′UTR-A_25_ or pDHAV-3′UTR-ΔA_25_ group were significantly higher than that in the pDHAV-Δ3′UTR-ΔA_25_ group, and the stimulation level in pDHAV-3′UTR-ΔA_25_ group was significantly higher compared to pDHAV-Δ3′UTR-A_25_ group (**Figure 6A**).

To further confirm the result, equal quantities of 0.8 μg of the *in vitro* transcribed products of the linearized plasmids pR-DHAV-Δ3′UTR-ΔA_25_, or pR-DHAV-Δ3′UTR-A_25_, or pR-DHAV-3′UTR-ΔA_25_, or pR-DHAV-1 were transfected into DEFs, and the cell lysates were separately collected at 4 hpt for western blot analysis. As observed for the reporter RNAs, significantly lower viral expression levels were observed in the pR-DHAV-Δ3′UTR-A_25_ and pR-DHAV-3′UTR-ΔA_25_ groups (**Figure 6B**) compared to pR-DHAV-1 group. Moreover, viral protein expression levels in the pR-DHAV-Δ3′UTR-A_25_ and pR-DHAV-3′UTR-ΔA_25_ groups were much higher than that in the pR-DHAV-Δ3′UTR-ΔA_25_ group (**Figure 6B**).

Based on the RNA stability result that deletion of 3′ UTR could lead into 31% and 43% decrement on RNA stability in pR-DHAV-Δ3′UTR-A_25_ and pDHAV-Δ3′UTR-A_25_ group (**Figure 2B**), as much as 77 and 82% decrement on viral protein or FLuc expression level was observed respectively, suggested that 3′ UTR strongly stimulate IRES-mediated translation efficiency; Besides, expression level in pR-DHAV-Δ3′UTR-A_25_ and pDHAV-Δ3′UTR-A_25_ group was higher compared to pR-DHAV-Δ3′UTR-ΔA_25_ and pDHAV-Δ3′UTR-ΔA_25_ group, indicating that poly(A)_25_ tail could function as an individual element to facilitate IRES-mediated translation. Moreover, removal of poly(A) tail could result in approximately a 50% decrease in both RNA stability and protein expression level (**Figures 2B, 6B**), which suggested that the decrement on viral protein or FLuc expression level might be attributed to the decline of RNA stability; The expression level in pR-DHAV-3′UTR-ΔA_25_ and pDHAV-3′UTR-A_25_ group was significantly higher compared to pR-DHAV-Δ3′UTR-ΔA_25_ and pDHAV-Δ3′UTR-ΔA_25_ group, indicated that 3′ UTR could individually and strongly stimulate IRES-mediated translation, and stimulatory effect on IRES-mediated translation derived by 3′ UTR was more efficient compared to poly(A)_25_ tail.

### Effects of Poly(A) Tail Length on IRES-Mediated Translation

**FIGURE 7:**
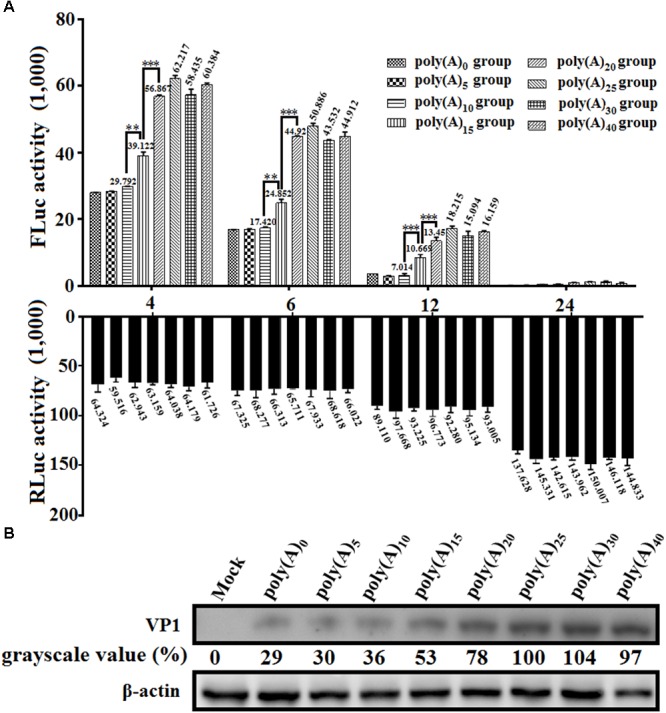
The effects of poly(A) tail length on IRES-mediated translation efficiency. **(A)** *In vitro* transcribed RNAs (0.8 μg) derived from linearized monocistronic reporter plasmids pDHAV-1-An containing various lengths of the poly(A) tail (n: 0, 5, 10, 15, 20, 25, 30, and 40) were co-transfected into DEFs with 0.1 μg of pGMLR-TK plasmid. Cell lysates were collected at 4, 6, 12, and 24 hpt to measure FLuc/RLuc activity. Columns and bars represent means and standard deviations, respectively, of three independent transfections. ^∗∗∗^*P* < 0.001, ^∗∗^*P* < 0.01. **(B)** The RNA-launched infectious clone pR-DHAV-1-An was transfected into DEFs, and the cell lysates were collected at 4 hpt for western blot analysis with anti-DHAV-1 monoclonal antibody 4F8 and HRP-labeled goat anti-mouse antibody. The grayscale value in poly(A)_25_ group was set as 100%.

To investigate the effects of poly(A) tail length on IRES-mediated translation efficiency, a series of monocistronic reporter plasmids contains various lengths of poly(A) tail (0, 5, 10, 15, 20, 25, 30, and 40 adenosine nucleotides) were constructed and named pDHAV-3′UTR-A_n_. The *in vitro* transcribed products of pDHAV-3′UTR-A_n_ were co-transfected into DEFs with 0.1 μg of pGMLR-TK plasmid, and the FLuc activities or RLuc activities were measured at from 4 to 24 hpt. The result showed that the FLuc expression level in the poly(A)_0-10_ group was significantly lower than that in the poly(A)_25_ group (**Figure 7A**). The decrease of FLuc expression in the poly(A)_0-5_ group could be partially due to the instability of the viral RNA. Since poly(A)_10_ reporter RNA shared similar stability with poly(A)_15-40_ group (**Figure 2A**), the result suggested that the deficiency of viral translation in poly (A)_10_ group is a not an indirect consequence of RNA degradation and poly(A)_10_ was not long enough to enhance IRES-mediated translation when present together with the 3′ UTR. Varying poly(A) tail length from 20 to 40 nucleotides had no effect on IRES-mediated translation efficiency, whereas increasing the length of the poly(A) tail from 15 to 20 nucleotides resulted in a ∼1.45-fold (*P <* 0.001) increase in FLuc activity, indicating that at least 20 adenines of the poly(A) tail are required for the optimal viral translation (**Figure 7A**).

To examine the viral protein expression with different poly(A) tail length, the mutated RNA-launched infectious clones pR-DHAV-1-An (*n*: 0, 5, 10, 15, 20, 25, 30, and 40) were transfected into DEFs, followed by collection of cell lysates and western blot analysis at 4 hpt. The poly(A)_0_, poly(A)_5_ and poly(A)_10_ groups showed equally low viral protein-expression level, and increasing poly(A) tail length from 15 to 25 could lead into consistently and significantly increment on viral expression level. However, no difference was observed in the remaining groups (poly(A)_25_, poly(A)_30_, and poly(A)_40_) (**Figure 7B**). Since poly(A)_10_ and poly(A)_15_ reporter RNA shared similar stability with poly(A)_20-40_ group (**Figure 2A**), the observed increase in translation efficiency was the result of a direct effect of poly(A) tail length and was not an indirect consequence of a change in RNA stability.

### The Effect of 3′ UTR and Poly(A) Tail on IRES-Mediated Translation Efficiency Is Cell-Line-Specific

**FIGURE 8:**
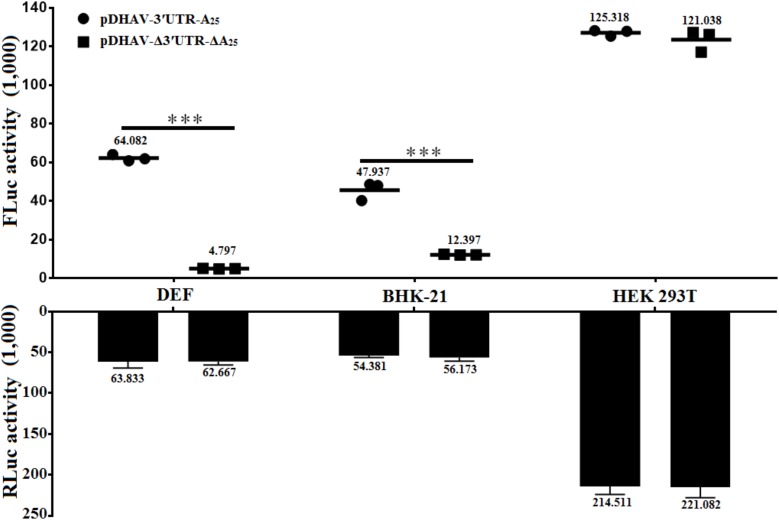
Effects of 3′ UTR plus poly(A) tail on IRES-mediated translation efficiency in various cell lines. Equal amount (∼2 × 10^5^) of DEFs, or BHK-21, or HEK 293T were seeded into 24-well plate 12 h before transfection. 0.8 μg of the Fluc activity of the pDHAV-Δ3′UTR-ΔA_25_ or pDHAV-3′UTR-A_25_ were co-transfected into DEFs, BHK-21 cells, and HEK 293T cells with 0.1 μg of pGMLR-TK plasmid to measure the FLuc/RLuc activity at 4 hpt. Data represent three replicate experiments for each group. ^∗∗∗^*P <* 0.001.

To determine whether the stimulatory effect of the 3′ UTR is cell-line- or species-specific, equal quantities of reporter RNAs from pDHAV-Δ3′UTR-ΔA_25_ or pDHAV-3′UTR-A_25_ were co-transfected into DEFs, or BHK-21, or HEK 293T cells with 0.1 μg of pGMLR-TK plasmid, and FLuc activity or RLuc activity was measured at 4 hpt. The 3′ UTR and poly(A) tail-mediated stimulation on IRES-mediated translation efficiency were only 1.2-fold (*P <* 0.05) in the HEK 293T cell line and 3.8-fold (*P <* 0.001) in the BHK-21 cell line, but 12.6-fold (*P <* 0.001) in DEFs (**Figure 8**). The collected data suggested that the IRES-mediated translation stimulation derived by 3′ UTR and poly(A) tail is specific to cell line.

## Discussion

The 5′ and 3′ terminal cis-acting elements within mRNAs play critical roles in gene expression by regulating translation efficiency. In conventional mRNAs, the 5′ cap and 3′ poly(A) tail provide anchors for a ribonucleoprotein (RNP) bridge that activates translation (Gallie, 1991). A circular RNP complex formed by the 5′ and 3′ ends of RNA and including the poly (A) binding protein (PABP) bind to the poly(A) tail regulates RNA replication during poliovirus negative-strand RNA synthesis (Silvestri et al., 2006). Increasing the length of the poly(A) tail from (A)_12_ to (A)_13_ could result in a dramatic 10-fold enhancement of negative-strand RNA synthesis during PV replication (Silvestri et al., 2006). In this research, DHAV-1 viral RNA replication was strongly influenced by the number of adenine bases (**Figure 3**), and DHAV-1 viral copy number increased significantly when the length of the poly(A) tail increased from 10 to 15 adenines or from 15 to 20 adenines (**Figure 3**). Based on the RNA stability result that poly(A)_10_ tail, and poly(A)_15_ tail and poly(A)_20_ viral RNA shared similar stability, it was confirmed that the change of viral genome copy number is due to the direct regulation of viral RNA replication by poly (A) tail. It has been demonstrated that the 3′ UTR or poly(A) tail of *picornaviruses* is important for maintaining the viral genome replication and translation efficiency (Rohll et al., 1995; Todd et al., 1997; Dobrikova et al., 2006). For example, IRES-driven translation is stimulated separately by the FMDV 3′ UTR and poly(A) sequences (Lopez de Quinto et al., 2002), deletion or substitution of FMDV 3′ UTR lead into abrogation in infectivity and virus replication (Saiz et al., 2001). However, to PV, the 3′ UTR was not absolutely essential for viral replication, and mutated PV without the complete 3′ UTR only exhibited a moderate deficiency in RNA synthesis (Brown et al., 2005). A previous study also showed that DHAV-1 translation initiation was 3′ UTR-independent (Liang et al., 2015). Nevertheless, in the present study, we showed that viral DHAV-1 viral RNA lacking the complete 3′ UTR was unable to replicate in DEFs (**Figure 4A**). This data might indicate that 3′ UTR was essential for viral replication, but also could indicated that viral RNA absent of 3′ UTR completely degraded from 4 hpt to 12 hpt. To measure the functional role of 3′ UTR at the same RNA stability level, the 3′ UTR was replaced by reverse complementary sequence of 3′ UTR to construct the mutated infectious clone, and the significant decrement on viral copy numbers in pR-DHAV-R3′UTR-A_25_ group suggested that 3′ UTR was essential for viral replication (**Figure 4C**). Moreover, we demonstrated that 3′ UTR and poly(A) tail were important for maintaining the RNA stability, and the length of poly(A) tail also affected RNA stability. Quantitation of the labeled input RNAs demonstrated that 41–53% of the poly(A)_0-5_ viral RNA remained intact at 4 h, compared to 95% for poly(A)_10_ viral RNA. Therefore, poly(A)_5_ tail was not long enough to maintain the RNA stability.

To *picornaviruses*, translation stimulated by the PABP might serve as a functional indication of mRNA integrity, given that only intact mRNAs with an intact 3′ region can be translated efficiently (Niepmann, 2009). For example, 3′ end of FMDV specifically interact with IRES element to control critical steps of the viral cycle (Serrano et al., 2006; Diaz-Toledano et al., 2017). To hepatitis C virus (HCV) of *flaviviridae*, 3′ UTR or a 3′ poly(A) tract of sufficient length promote efficient translation right after the initiation phase (Bradrick et al., 2006; Song et al., 2006; Diaz-Toledano et al., 2017; Niepmann et al., 2018). However, it has not been confirmed whether the interaction between the poly(A) tail of DHAV-1 and PABP stimulates cap-dependent translation because the “closed loop” allows ribosomes to recycle more easily from the mRNA 3′ end to its 5′ end. In this study, we investigated 3′ UTR plus poly(A)_25_ tail strongly enhanced IRES-mediated translation efficiency. During the viral propagation of DHAV-1, it was believed that PABP specifically binds to poly(A) tail and interact with eIF4G to stimulate *picornavirus* translation (Svitkin et al., 2001). However, the *RLuc* gene was placed in front of 5′ UTR in their research, which might interference the interaction between poly(A) tail and 5′ UTR because both the 5′ and 3′ ends of RNA were involved to form a circular RNP complex for *picornaviruses* members (Silvestri et al., 2006). Further investigations about whether the additional nucleotide in front of 5′ UTR affects IRES-mediated translation efficiency are necessary.

The poly(A) tail is the terminal structure of the viral genome and plays a critical role in efficient initiation of negative-strand RNA synthesis (Melchers et al., 1997). To HCV, the 3′ UTR and poly(A)_50_ tail interchangeably improved translation efficiency dependent upon the HCV IRES, and the poor translation efficiency of reporter RNAs lacking the 3′ UTR or poly(A)_25_ tail might reflect inefficient release of ribosomes that terminate aberrantly (Bradrick et al., 2006). In this research, the 3′ UTR or poly(A)_25_ tail separately enhanced DHAV-1 IRES-mediated translation efficiency, and that the length of the poly(A) tail strongly influenced translation efficiency (**Figures 6, 7**). Removal of the 3′ UTR, or the poly(A) tail, or both the 3′ UTR and poly(A) tail could result in reduction on the efficiency of DHAV-1 translation and impairment on viral growth. Analysis of the RNA stability levels of the reporter or viral RNAs present in transfected DEFs indicated that stimulation of relative IRES activity was not the consequence of a higher stability of the corresponding transcripts (**Figure 2**). This effect was magnified by using the monocistronic reporter system of DHAV-1 (**Figures 5, 6** and **7**). IRES-mediated *picornavirus* mRNA translation is stimulated by the poly(A) tail, and the interaction between eIF4G and PABP improves IRES-mediated translation during viral replication of PV (Svitkin et al., 2007). The monocistronic reporter system and DHAV-1 RNA-launched infectious clones harboring a poly(A)_0-10_ tail exerted no stimulatory effect on IRES-mediated translation efficiency, which might be due to that the poly(A)_0-10_ tail is not long enough to bind PABP, resulting in failure to create a closed-loop complex between eIF4G and PABP.

DHAV-1 replicates preferentially in DEFs (peak: 10^6.31^ copies/μL) and can replicate in BHK-21 cells (peak: 10^4.17^ copies/μL); however, it cannot replicate in HEK 293T cells. In this study, the translation enhancement derived by the 3′ UTR and poly(A) tail of DHAV-1 in DEFs was much stronger than in BHK-21 (**Figure 8**), indicating that the stimulatory role of 3′ UTR and poly(A) tail on IRES-mediated translation efficiency was cell-line-specific. However, no significant difference was observed in HEK 293T group, which may due to the fact that the HEK 293T cells effectively degrade non-sense mediated (NMD) decay targets when reporter RNAs are produced from extrachromosomal plasmid DNA (Gerbracht et al., 2017). Additionally, it has been reported that differential NMD efficiency was observed in HEK 293T cells depending on whether stable or transient transfection have been performed (Gerbracht et al., 2017), indicating that the equal expression level between complete and truncated reporter RNAs may be due to the degradation on RNA stability. To further explore the exact mechanism during DHAV-1 replication, identification of the cellular/viral factors that affect DHAV-1 IRES-mediated translation and viral replication are necessary.

## Author Contributions

J-HC, F-CL, and S-JJ designed the experiments. J-HC, R-HZ, P-FL, J-JL, and S-SS performed the experiments. J-HC, R-HZ, S-LL, J-MG, YW, Z-JX, and S-JJ analyzed the data. J-HC, S-LL, and S-JJ wrote the manuscript.

## Conflict of Interest Statement

The authors declare that the research was conducted in the absence of any commercial or financial relationships that could be construed as a potential conflict of interest.
